# Fall Prevention Model at Local Non-Profit Equipment Exchange Program Utilizing the STEADI: a Compilation of Two Pilot Studies

**DOI:** 10.1093/geroni/igad104.2346

**Published:** 2023-12-21

**Authors:** Constance Lewis, Karen Probst, Rachel Tanilli, Alix Schoenfeld

**Affiliations:** Gannon University, Erie, Pennsylvania, United States; Gannon University, Erie, Pennsylvania, United States; Gannon University, Erie, Pennsylvania, United States; Gannon University, Erie, Pennsylvania, United States

## Abstract

Individuals picking up gently used durable medical equipment (DME) from a local non-profit equipment exchange program in Erie, Pennsylvania were prompted with the CDC’s Stopping Elderly Accidents, Deaths & Injuries (STEADI) 3-item abbreviated questionnaire and contacted by telephone 6 months later to assess if this survey tool can accurately predict falls. Individuals were also given a STEADI pamphlet upon equipment pickup to educate community members on fall risk. Ninety individuals, who ranged from caregivers to the individual themselves, chose to participate in the follow-up research phone call. Our researchers found that while the abbreviated 3-item STEADI suggested that 80% of community members (in need of DME by way of an equipment exchange program) were predicted to be at risk for a fall, only 22.5% suffered a fall within a six-month follow-up period. This questions the validity of the 3-item abbreviated STEADI, though additional studies are recommended to improve the participant percentage (only 90 out of 255 individuals, or 35%, answered the follow-up call questions) as well as increasing the length of wait time to assess for subsequent fall incident beyond six months. Additionally, only 34% of individuals recalled receiving a pamphlet when picking up equipment, and of those 31 individuals, only 10 of them (32%) remembered what the pamphlet said. This suggests that the simple use of a brochure handout to decrease fall risk amongst community-dwelling adults does not seem to be an effective educational strategy within the Erie, PA community.

